# Optimization of Magnesium Potassium Phosphate Cements Using Ultrafine Fly Ash and Fly Ash

**DOI:** 10.1021/acssuschemeng.2c04987

**Published:** 2023-02-10

**Authors:** Yongshan Tan, Laura J. Gardner, Brant Walkley, Oday H. Hussein, Hao Ding, Shikuan Sun, Hongfa Yu, Neil C. Hyatt

**Affiliations:** † College of Civil Science and Engineering, Yangzhou University, Yangzhou 225127, China; ‡ NucleUS Immobilisation Science Laboratory, Department of Materials Science and Engineering, University of Sheffield, Sheffield S1 3JD, UK; § Department of Chemical and Biological Engineering, University of Sheffield, Sheffield S1 3JD, UK; ∥ Department of Civil and Airport Engineering, Nanjing University of Aeronautics and Astronautics, Nanjing 210016, China; ⊥ School of Material Science and Energy Engineering, Foshan University, Foshan, Guangdong 528000, China

**Keywords:** magnesium potassium phosphate cement, fly ash, characterization, particle size distribution

## Abstract

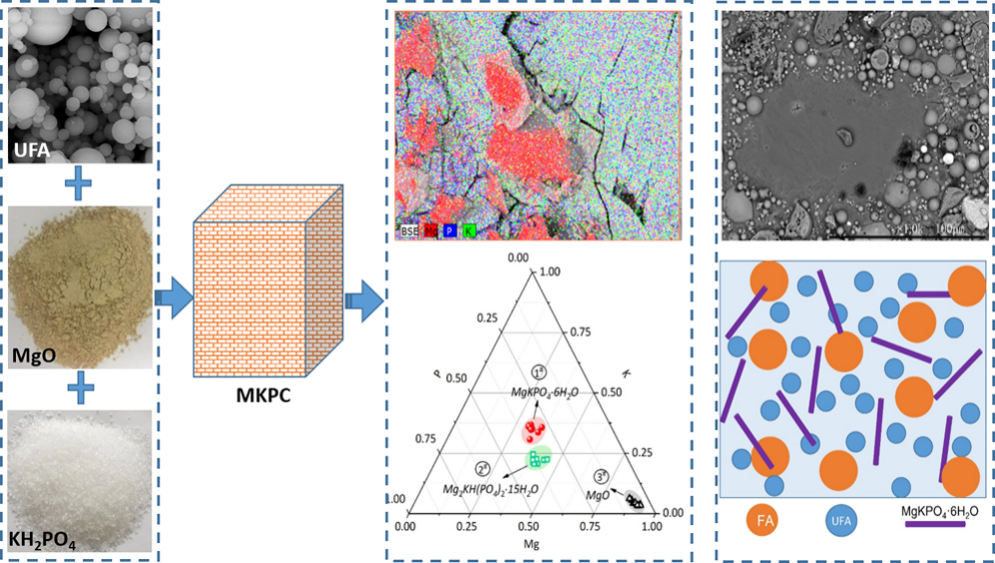

The effect of ultrafine fly ash (UFA) and fly ash (FA) on the physical properties, phase assemblage, and microstructure of magnesium potassium phosphate cement (MKPC) was investigated. This study revealed that the UFA addition does not affect the calorimetry hydration peak associated with MKPC formation when normalized to the reactive components (MgO and KH_2_PO_4_). However, there is an indication that greater UFA additions lead to an increased reaction duration, suggesting the potential formation of secondary reaction products. The addition of a UFA:FA blend can delay the hydration and the setting time of MKPC, enhancing workability. MgKPO_4_·6H_2_O was the main crystalline phase observed in all systems; however, at low replacement levels in the UFA-only system (<30 wt %), Mg_2_KH(PO_4_)_2_·15H_2_O was also observed by XRD, SEM/EDS, TGA, and NMR (^31^P MAS, ^1^H-^31^P CP MAS). Detailed SEM/EDS and MAS NMR investigations (^27^Al, ^29^Si, ^31^P) demonstrated that the role of UFA and UFA:FA was mainly as a filler and diluent. Overall, the optimized formulation was determined to contain 40 wt % fly ash (10 wt % UFA and 30 wt % FA (U10F30)), which achieved the highest compressive strength and fluidity and produced a dense microstructure.

## Abstract 2

The mixed addition of UFA and FA (optimized formulation is the U10FA30 addition) drives MKPC to have the highest compressive strength, highest workable paste with a dense microstructure.

## Introduction

As of 2019, the UK is forecast to produce 4.42 million cubic meters of radioactive wastes as a result of nuclear decommissioning (including estimated future arisings up to 2135),^1^ which needs to be safely treated and disposed in order to reduce the associated hazards while minimizing environmental impact. This volume accounts for all wastes: high-level (HLW), intermediate-level (ILW), and low-level (LLW) wastes generated from civil nuclear power stations, in addition to contributions from the medical and defense sectors.^1^ At present, Portland cement is the main encapsulation material used for ILW in the UK, which accounts for 4.9% and 5.4% of the total radioactivity and volume, respectively, and consists of the following: fuel cladding, graphite, plutonium-contaminated wastes, contaminated metals, and sludges.^1^

When compared with alternative stabilization and solidification methods such as glass immobilization and synroc/ceramic immobilization, cementation is a simple process and has low cost with high sorption or uptake capacity for many contaminants.^2^ Therefore, Portland cement (PC) has been widely used as an immobilization agent for radioactive waste and hazardous wastes (e.g., heavy metals).^3^ However, PC with a high pH and free-water content is not a universal encapsulation grout suitable for the wide variety of ILW wastes requiring conditioning. For acidic radioactive wastes, it must be neutralized before it can be solidified with cement, which increases the processing costs.^4^ In PC composite grouts with slag replacement (up to 90 wt %), the basicity of the system remains at pH ∼12.5, which could promote corrosion of reactive metals (e.g., Mg in Magnox swarf, Al, and U) present in intermediate- and low-level radioactive wastes. Corrosion results in the formation of expansive phases and hydrogen (e.g., Mg_(s)_ + 2H_2_O_(l)_ → Mg (OH)_2(s)_ + H_2(g)_), which could result in stress-induced cracking that could ultimately damage the conditioned wasteform.^5−7^ Therefore, the selection of an appropriate cement system based on waste characteristics is important to provide long-term waste form stability, frequently referred to as a toolbox for nuclear waste management.^8^

Magnesium phosphate cement (MPC) is an inorganic cementitious material, which is classified as a low-temperature ceramic material sometimes referred to as chemically bonded ceramics (CBCs), which are formed *via* an acid–base reaction between MgO and a soluble acid phosphate (typically an ammonium or potassium phosphate), resulting in the formation of magnesium phosphate salt (MgNH_4_PO_4_·6H_2_O or MgKPO_4_·6H_2_O, respectively) with cementitious properties, shown in eqs 1 and 2. When compared with Portland cement, MPC has many advantages, which include the following: fast setting and hardening; high early strength, with 20 and 40 MPa achievable after 1 and 3 h curing, respectively; fast setting including in low-temperature scenarios (5 to −20 °C), high bonding strength with old concrete and mortars; and good abrasion resistance and frost resistance.^9−11^ Due to the above properties, MPC is widely used as building materials for rapid repair of roads, bridges, and airport runways. In addition, MPC can be used to immobilize industrial wastes, toxic heavy metals, and radioactive wastes; however, it should be noted that magnesium potassium phosphate cement (MKPC; using KH_2_PO_4_ as the phosphate source) is often favored for these applications owing to the liberation of NH_4_ if struvite is the binder phase, which can occur during setting and at temperature >50 °C.^12−15^
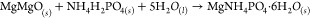
1
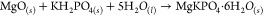
2

Due to the dense structure and physical and chemical stability of MKPC, many published articles^5,16−19^ have confirmed that MKPC has excellent solidification and storage capacity for radioactive waste. When compared with ordinary Portland cement (PC), MKPC had good compatibility with respect to corrosion rates (i.e., H_2_ gas generation) and microstructural changes. Successful demonstrations of MKPC as an encapsulating grout were partially derived from the lower pH (compared to PC blends) that can inhibit or passivate uranium corrosion.

In order to reduce the heat of hydration, water-cement ratio, and costs of MKPC, several investigations have considered the effect of fly ash (FA) on MKPC performance and the acid–base hydration mechanism. Yang *et al.*(20) and Li *et al.*(21) studied the effect of FA on the durability of MKPC in water and salt solutions (sulfate and chloride) and found that the incorporation of fly ash can improve the mechanical properties of MKPC and the pore structure, leading to an improved durability of MKPC with fly ash. Xu *et al.*(22) found that the addition of high CaO content of FA, up to 50 wt %, can significantly improve the compressive strength of MKPC. As the fly ash content exceeded 50 wt %, the observed decrease in MKPC strength may be attributed to the formation of CaK_3_H(PO_4_)_2_ and Mg_3_(PO_4_)_2_·22H_2_O. Mo *et al*.^23^ demonstrated that the addition of FA can improve the pore structure of MKPC. Gardner *et al.*(9) found that FA (or slag) additions at 50 wt % (based on the sum of MgO, KH_2_PO_4_, and H_2_O) resulted in a compact microstructure of the MKPC hardened matrix with an improved mechanical strength at a water-to-solids ratio of 0.24. The utilization of ^27^Al, ^29^Si, and ^39^K solid-state NMR at this 0.24 w/s revealed the presence of a secondary potassium aluminosilicate phase, which could have only resulted from the partial dissolution and reaction of FA (or slag). In summary, FA incorporation can significantly improve various properties of MKPC, including improving the workability, mechanical properties, and durability performance of MKPC, reducing the heat of hydration of MKPC and extending the setting time of MKPC. In addition, FA as an industrial byproduct can significantly reduce the cost of MKPC without deleterious effect on the mechanical performance.

With the development of modern civil engineering and environmental engineering, higher requirements are put forward for the mechanical properties and durability of cementitious materials. Based on this, scholars have prepared ultrafine fly ash (UFA) by using a new process based on fly ash. UFA is the second superfine grinding product of fly ash, a byproduct from thermal power plants. The ultrafine grinding of fly ash releases the smaller microspheres contained in the larger/hollow fly ash particles.^24^ At present, UFA is widely used as a mineral admixture in high-performance concrete.^25,26^ UFA is an ideal material to utilize due to its smaller particles, which when mixed with Portland cement can lead to the formation of ultrahigh-performance concretes^26^ and accelerated hydration.^27^ These systems were demonstrated to have an improved filling effect associated with the fine particle size of UFA,^28^ leading to detectable improvements in the physical properties, microstructure, and durability.^27,29,30^ In geopolymers, the inclusion of UFA also leads to a reduced porosity.^31^ The above studies indicate that UFA could have better improvement on the properties of cementitious materials compared to FA. Combined with the above literature review, it is found that for MKPC systems, more reports focus on the study of the effect of FA on the performance of MKPC. Therefore, in order to optimize the performance and microstructure of MKPC, it is necessary to also investigate the effects of UFA and FA/UFA blends on the performance and microstructure of MKPC as an encapsulating grout.

In the present work, UFA was incorporated into MKPC pastes *via* two different design methods to optimize the diluent source (s) and replacement level in order to improve the properties of blended MKPCs. First, the effect of UFA inclusion within MKPC was investigated at different replacement ratios, 0 to 40 wt %. Second, the effect of a blended composite (UFA and FA) at varying ratios was determined on MKPCs, where the total replacement was fixed at 40 wt % with the aim of utilizing the characteristics of each diluent source to improve the overall properties of MKPCs. The UFA/MKPC and UFA:FA/MKPC blended pastes were systematic evaluated using setting time, compressive strength, X-ray diffraction (XRD), thermogravimetry (TGA), scanning electron microscopy with energy-dispersive X-ray spectroscopy (SEM/EDX), mercury intrusion porosimetry (MIP), and solid-state MAS NMR (^27^Al, ^29^Si, ^31^P, ^1^H-^31^P cross polarization (CP) and ^1^H-^29^Si CP) spectroscopy techniques.

## Materials and Methods

### Raw Materials

**Figure 1:**
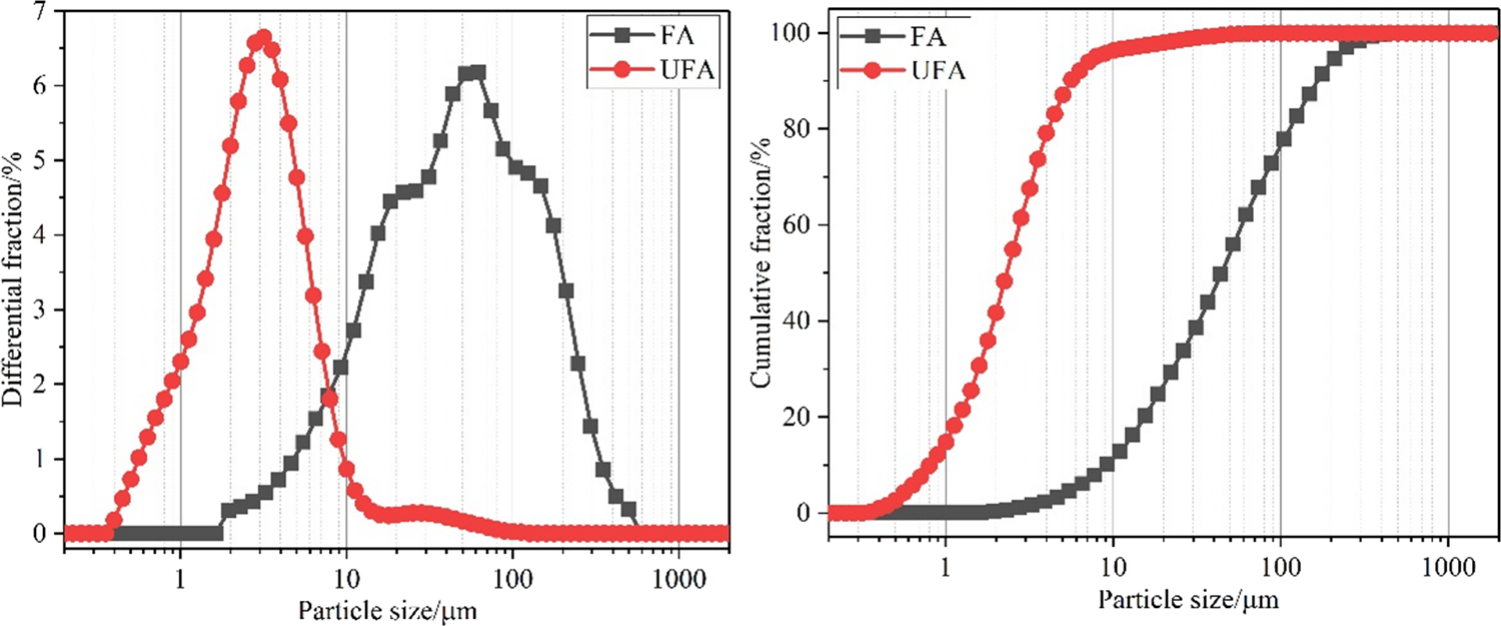
Particle size distribution of UFA and FA.

Dead burnt magnesia (MgO) at 90% purity was obtained from Richard Baker Harrison Ltd. Mono potassium phosphate (KH_2_PO_4_) was provided by Prayon UK as Food Grade E340MKP, and the certificate of analysis purity was >99%. Ultrafine fly ash (UFA) was supplied by Shenzhen Daote Science and Technology Ltd., and fly ash (FA) was supplied by CEMEX as PFA BS EN 450-1S. Granular boric acid (H_3_BO_3_) was sourced from Fisher Scientific UK (CAS number 10043-35-3, laboratory-grade) with a purity of 99.5%. The chemical compositions of MgO, UFA, and FA determined by X-ray fluorescence (XRF) oxide analysis are reported in Table 1 while the particle size distributions of FA and UFA are displayed in Figure 1.

**Table 1:** Chemical Compositions of Raw Materials (wt %)

raw materials	CaO	Al_2_O_3_	SiO_2_	Fe_2_O_3_	MgO	K_2_O	Na_2_O	TiO_2_	P_2_O_5_
MgO^9^	2.1	1.7	4.3	1.5	88.9	0.1	<0.1		<0.1
FA^9^	2.4	25.2	50.2	9.3	1.7	3.6	1.1		0.3
UFA	17.6	6.8	65.7	0.1	0.1	<0.1	<0.1	<0.1	<0.1

### Mix Design

The mix proportions of the investigated MKPC pastes are listed in Table 3. In order to ensure the workability of MKPC paste, boric acid (H_3_BO_3_) was used as a set retarder in the MKPC paste at a weight ratio of H_3_BO_3_ to MgO was 8:100 (i.e., 8 wt % MgO content). The molar ratio of MgO:KH_2_PO_4_ (M/P) was 2:1, and the water-to-solids (w/s) ratio was 0.22 (by weight of MgO and KH_2_PO_4_). UFA or FA was incorporated in the MKPC pastes through two different design methods:

(1) The addition of UFA as a diluent (by weight of MgO and KH_2_PO_4_) at the following increments: 0 wt % (control), 10 wt %, 20 wt %, 30 wt %, and 40 wt %. The objective for this design was to understand the effects of an increasing UFA content on the mechanical properties and phase assemblage.

(2) The addition of blended fly ash sources (UFA and FA) at varying replacement levels were produced at a fixed total diluent addition (40 wt %), and the UFA:FA ratios were: 40:0, 30:10, 20:20, 10:30, and 0:40 wt %. The purpose for this experiment was to improve the mechanical properties of MKPCs by utilizing the different particle sizes of UFA and FA.

The precursors (MgO, KH_2_PO_4_, H_2_O, UFA/FA, and H_3_BO_3_) were mixed in an Heidolph RZR 2020 overhead stirrer at speed 500 rpm for 5 min, ensuring all components were mixed thoroughly before casting into 15 mL centrifuge tubes or used directly (for studies of setting time, calorimetry, mini-slump). For compressive strength cubes, the precursors (MgO, KH_2_PO_4_, UFA/FA, and H_3_BO_3_) were mixed for 10 min in a Kenwood benchtop mixer at low speed. Afterward, the pastes were cast into 50 × 50 × 50 mm steel cube molds. All cast samples were cured in an environmental chamber at 20 °C and 95% relative humidity until testing at 3, 7, and 28 days of curing.

### Analytical Methods

Setting time was characterized on wet pastes by automatic Vicat apparatus, which was measured for 90 penetrations, with 1 penetration set for every 5 min at room temperature (20 ± 3 °C). The hydration heat flow of all samples were monitored by isothermal conduction calorimetry (TA instruments TAM Air) at 20 °C. Each formulation was prepared according to Table 2, and the dry precursors (MgO H_3_BO_3_, KH_2_PO_4_, and UFA/FA) were weighed into a plastic ampoule and well mixed. Deionized (DI) water was added and mixed for ∼2 min until a homogeneous paste was formed, after which 20 g was weighed into a plastic ampoule and loaded into the calorimeter, alongside a reference ampoule (containing an equivalent quantity of DI water). Mini-slump measurements were performed using a scaled down Abrams cones (*h*: 57 mm, *d* (top): 19 mm, and *d* (bottom): 38 mm)^32,33^ and a poly(methyl methacrylate) sheet marked with 20 × 20 mm grid squares. A photograph was taken of the final slump from directly above the sample, and the slump area was then calculated using ImageJ software^34^ calibrated to the grid squares. The values reported for each formulation correspond to an average of 3 measurements, with the calculated error bars equivalent to ±1 standard deviation.

**Table 2:** Particle Size Distribution of FA and UFA (Errors Are Reported to ±1 S.D.)

raw materials	*d*_10_ (μm)	*d*_50_ (μm)	*d*_90_ (μm)
FA	2.70 ± 0.19	14.00 ± 0.30	66.1 ± 0.35
UFA	0.94 ± 0.01	2.69 ± 0.12	6.52 ± 0.27

**Table 3:** Mix Proportions of the MKPC Pastes

formulation	MgO (g)	KH_2_PO_4_ (g)	UFA (g)	FA (g)	H_2_O (g)	H_3_BO_3_ (g)
control	37.05	62.95	0	0	22	2.97
U10	33.35	56.65	10	0	22	2.67
U20	29.65	50.35	20	0	22	2.37
U30	25.95	44.05	30	0	22	2.08
U40	22.23	37.77	40	0	22	1.78
U30F10	22.23	37.77	30	10	22	1.78
U20F20	22.23	37.77	20	20	22	1.78
U10F30	22.23	37.77	10	30	22	1.78
F40	22.23	37.77	0	40	22	1.78

Compressive strength was determined from triplicate 50 mm cube specimens after 3, 7, and 28 days of curing using a Controls Automax 5.0 machine at a loading rate of 0.25 MPa/s. The crystalline phases of the MKPC pastes were identified by X-ray powder diffraction (XRD) using a Bruker D2 Phaser diffractometer with a Lynxeye detector and Ni filtered Cu Kα radiation (λ = 1.5418 Å) at 30 kV and 10 mA. Diffraction patterns were collected between 5° < 2θ < 60° with a step size of 0.02° and 1 s per step. Thermogravimetric analysis (TGA) was performed using a Perkin Elmer TGA 4000 instrument coupled with a Hiden mass spectrometer between 30 and 1000 °C at a heating rate of 3 °C/min, under nitrogen flowing at 20 mL/min. For XRD and TGA analysis, a representative sample was ground into a diner powder and sieved using a 75 μm brass sieve.

After 28 days of curing, microstructures were observed using a Hitachi TM3030 scanning electron microscope (SEM) coupled with a Bruker Quantax 70 energy-dispersive X-ray spectroscopy system (EDX) at a working distance of 8 mm using a silicon drift detector. SEM samples were prepared by cold epoxy resin mounting (cured for 24 h), ground, polished to a 1 μm diamond finish, and carbon-coated. For pore size distribution and porosity analysis, samples were crushed into small pieces after 28 days of curing and dried at 60 °C for 4 h. Mercury intrusion porosimetry (MIP) analysis was then performed using a Micromeritics Autopore V 9600. The maximum pressure applied was 208 MPa, the surface tension was 485 mN/m, and the contact angle was 130°.

Solid-state single pulse ^27^Al, ^29^Si, and ^31^P magic angle spinning (MAS) NMR data were acquired on a Bruker Avance III HD 500 spectrometer at 11.7 T (B_0_) using a 4.0 mm dual resonance CP/MAS probe, yielding a Larmor frequency of 130.32 MHz for ^27^Al, 99.35 MHz for ^29^Si, and 202.457 MHz for ^31^P. ^27^Al MAS NMR spectra were acquired using a 1.4 μs non-selective (π/2) excitation pulse, a measured 1 s relaxation delay, a total of 256 scans, and spinning at 12.5 kHz. ^29^Si MAS NMR spectra were acquired using a 4.0 μs non-selective (π/2) excitation pulse, a measured 60 s relaxation delay, a total of 256 scans, and spinning at 12.5 kHz. ^31^P MAS NMR spectra were acquired using a 2.5 μs non-selective (π/2) excitation pulse, a measured 30 s relaxation delay, a total of 64 scans, and spinning at 12.5 kHz. ^1^H-^29^Si cross-polarization (CP) MAS NMR experiments were performed using the same instrument with a spinning frequency of 12.5 kHz, ^29^Si non-selective (π/2) pulse width of 4.0 μs, initial ^1^H non-selective (π/2) pulse width of 2.5 μs, recycle delay of 1.5 s, and Hartmann–Hahn contact periods of 1.7 ms. A nominal ^1^H decoupling field strength of 80 kHz was employed during acquisition, and 5120 scans were collected per experiment. ^1^H-^31^P cross-polarization (CP) MAS NMR experiments were performed using the same instrument with a spinning frequency of 12.5 kHz, ^31^P non-selective (π/2) pulse width of 2.0 μs, initial ^1^H non-selective (π/2) pulse width of 2.5 μs, recycle delay of 1.5 s, and Hartmann–Hahn contact periods of 2.0 ms. A nominal ^1^H decoupling field strength of 80 kHz was employed during acquisition, and 256 scans were collected per experiment. All ^27^Al, ^29^Si, and ^31^P spectra were referenced to 1.0 M aqueous Al(NO_3_)_3_, pure tetramethylsilane (TMS), and 1.0 M aqueous H_3_PO_4(aq)_, respectively, at 0 ppm.

## Results and Discussion

### Setting Time and Mini-slump

**Figure 2:**
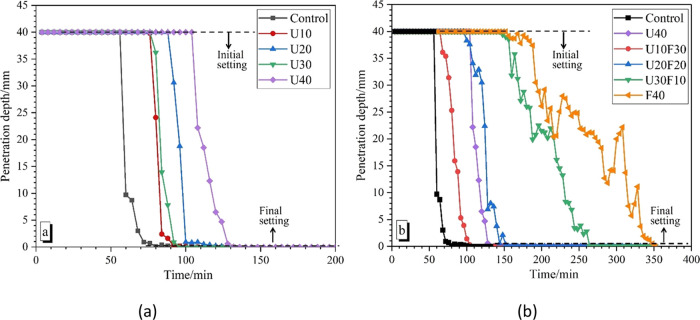
Setting time of MKPCs with (a) increasing UFA additions and (b) varying the ratio of UFA:FA at 40 wt % addition.

The penetration depth of Vicat measurements was used to indicate the setting time of MKPC pastes. Figure 2 shows the setting time results of MKPC pastes: when the penetration depth was below 40 mm, this represented the initial setting time of MKPC. When the penetration depth reached 0 mm, this indicated that the final setting time of MKPC was achieved.^35^ In Figure 2a, the addition of UFA with elevating content was observed to increase the setting time. For instance, when the dosage of UFA in the MKPC paste increased from 0 to 40 wt %, the initial setting time increased from 56 to 104 min, while the final setting time increased from 104 to 132 min, respectively.

In contrast, when the composite blend UFA:FA was utilized, the setting time of the MKPC paste increased significantly, as shown in Figure 2b. When the replacement addition of FA increased from 0 wt % (U40), 20 wt % (U20F20), and 40 wt % (F40), commensurate with a decrease in UFA content, the final setting time of the blended MKPC paste was extended from 132, 156, and 356 min, respectively. These results indicate that the addition of both UFA and FA can prolong the setting time and thus workability; however, the addition of FA had a greater impact on retarding the setting of MKPC pastes than UFA only. This was in agreement with the current literature, where the setting time of MKPC pastes increased at higher FA inclusions.^21,35,36^ It was determined that a FA replacement between 30 and 50 wt % will provide the best improvement on MKPC, with respect to extended workability.^37^ The delayed setting can be explained as follows: UFA and FA may act as inert fillers and diluent, which reduces bleed and increases the packing fraction of solid materials by occupying interstices between other particles. The physical effects of the UFA and FA may contribute to the extended setting time as the greater particle size of FA (*D*_50_ = 14.0 μm, Table 2) will occupy more space and result in further dilution of the MKPC precursors (compared to the finer particles of UFA where *D*_50_ = 2.69 μm, Table 2), which can contribute to the longer setting time of the MKPC paste when blended with FA.

**Figure 3:**
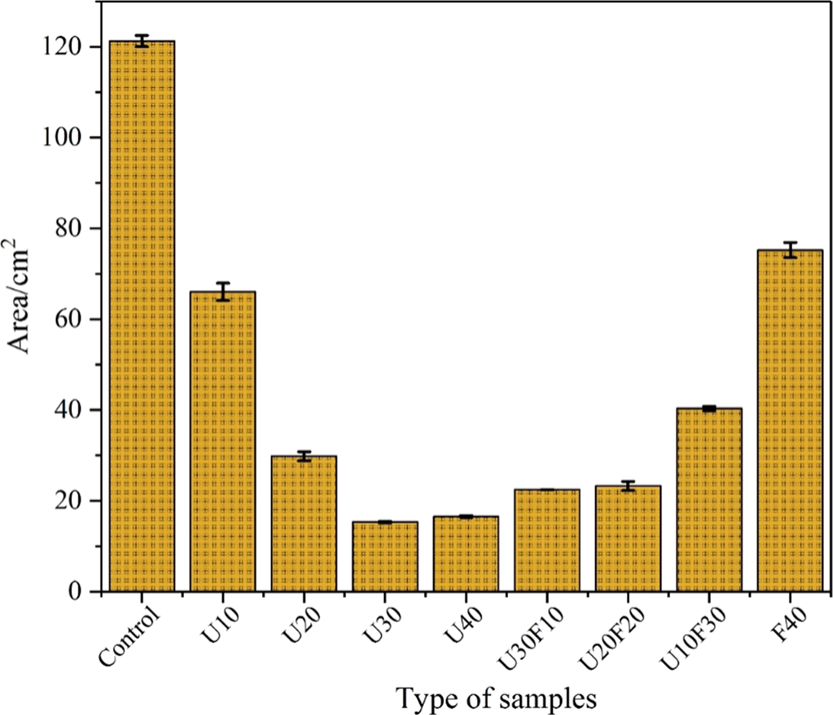
Miniature slump measurements for MKPC pastes with varying compositions.

In Figure 3, the miniature slump measurements for MKPC pastes with varying compositions are shown. The control MKPC (with no UFA or FA) achieved the highest miniature slump value at 122 cm^2^ without fillers that would be detrimental to overall workability of MKPC. The incorporation of fillers (UFA or FA) would significantly improve this defect. The miniature slump of the MKPC paste gradually decreased with an increasing UFA replacement dose. When the ratio reached to 30 and 40 wt %, the miniature slump of the MKPC paste reached the minimum value (15 ± 0.2 cm^2^). However, when the total replacement ratio of UFA:FA was fixed at 40 wt %, the opposite was true; the miniature slump gradually increased with the increasing FA replacement dose. This indicated that FA additions can significantly improve the workability of MKPC paste whereas UFA-only formulations can impede the fluidity (as discussed above). UFA, as a product of secondary grinding of FA, has a spherical structure like FA, where the spherical particle shape of FA acts to reduce the viscosity and yield stress of the fresh cement paste, known as the “ball-bearing” effect in the cement paste.^38^ This combined with the lower water demand of FA (compared to UFA) explains why the workability of the MKPC paste was significantly improved as the FA addition increased.^39^ In the literature, UFA has been shown to provide some improvement to the workability;^38^ however, the higher specific surface area of UFA (and therefore higher water demand) will have a negative impact on the workability of the fresh MKPC paste. Furthermore, in low-workable pastes, the overall water-to-cement ratio of MKPC was noted to decrease, which reduces the reaction rate of MgKPO_4_·6H_2_O and can lead to expansion issues at later ages, associated with the continued formation of MgKPO_4_·6H_2_O within a hardened binder.^40^ Therefore, it would not be appropriate to use UFA with high replacement dosage due to the poor workability and potential long-term stability issues; however, the use of a composite UFA-FA blend (U10F30) achieved better results (than UFA or FA only) when considering compressive strength and workability.

### Compressive Strength

**Figure 4:**
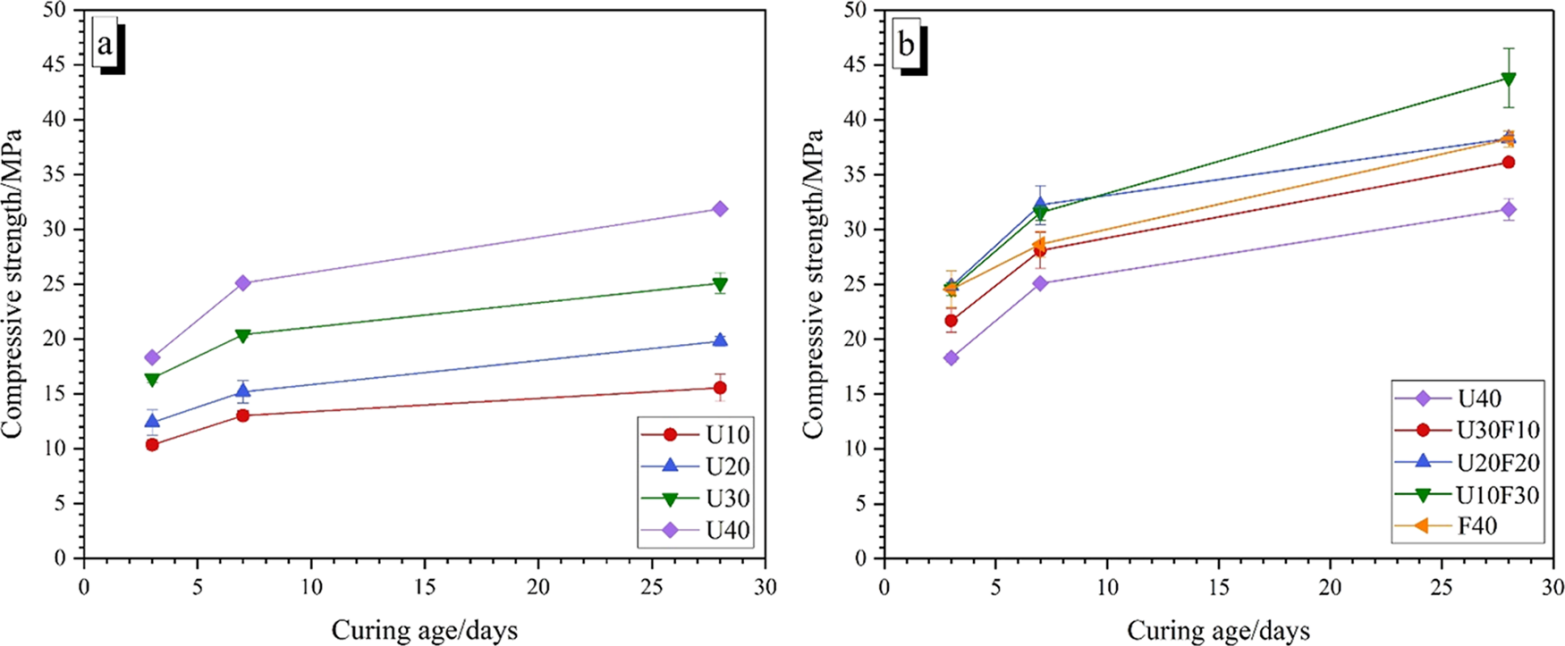
Compressive strength of MKPC pastes with (a) increasing UFA additions and (b) varying ratio of UFA/FA at 40 wt % addition.

The compressive strength development of the MKPC pastes with various formulations between 3 and 28 days of curing is shown in Figure 4. The data indicate that the compressive strength of blended MKPC pastes increased as the UFA content increased at all curing ages. The highest compressive strength (at 28 days) for the U40 wt % reached 31.8 ± 1.0 MPa. Compared with other MKPC systems blended with FA (albeit using slightly different Mg/P ratio and w/s ratios),^5,9^ UFA appears to enhance the compressive strength of MKPC by increasing the strength from ∼25 MPa at 28 days^5,9^ to ∼32 MPa in the present study. When the UFA replacement ratio is below 30 wt %, the compressive strength was relatively low (e.g., the sample of U30 achieved 12 and 20 MPa at 3 and 28 days, respectively). For the control, U10, U20, and U30 samples, cracks were visible on the surface of the cubes prior to measurements, which could be the main reason why the samples of U10, U20, and U30 achieved lower compressive strength values. The reason for this cracking may be caused by the low M/P molar ratio, and further investigations should be performed.

In the UFA:FA composite formulations, the compressive strength was observed to be higher than the UFA-only formulations. At 7 days, the compressive strength values for U20F20 and U10F30 were 32.3 ± 1.8 and 31.5 ± 0.7 MPa, respectively, which were ∼28% higher than the U40 sample (25.1 ± 0.2 MPa, 7 days). These compressive strength values revealed that the blended addition of UFA and FA was conducted to an improved strength development of MKPC.

### Isothermal Calorimetry

**Figure 5:**
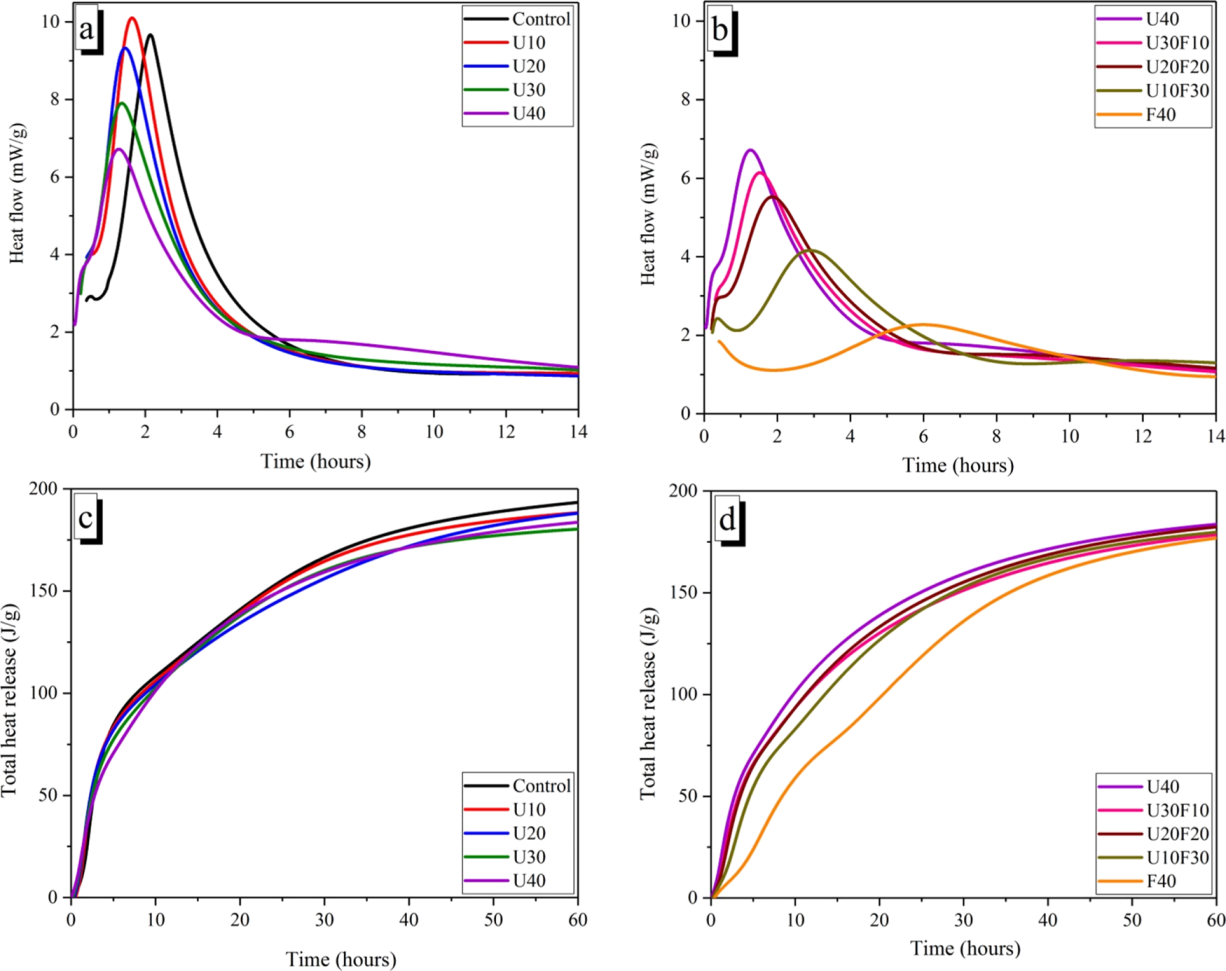
Isothermal calorimetry of the MKPC pastes displaying heat flow (a, b) and cumulative heat flow (c, d) for varying UFA additions and the varying ratio of UFA:FA fixed at 40 wt %.

Figure 5 shows the hydration heat release curves of the MKPC paste with different formulations. Two exothermic peaks appeared in the MKPC pastes with different formulations during the hydration process. In Figure 5, the appearance of the first exothermic peak (up to 1 h) represents multiple chemical reaction processes: first, when the MKPC precursors are mixed with water, KH_2_PO_4_ dissolves and quickly forms H_2_PO_4_^–^ and HPO_4_^–^, which makes the cement paste weakly acidic and subsequently induces the dissolution of MgO to form Mg^2+^, which is exothermic. HPO_4_^–^ and Mg^2+^ react to form amorphous and crystalline magnesium phosphates in an exothermic process that overlap with the continuing dissolution of MgO.^41,42^

The second exothermic peak occurring between 1 and 6 h was more intense, which was associated with the formation of amorphous hydration products, which begin to reach saturation and crystallize to form the hydration product, MgKPO_4_·6H_2_O.^43,44^

**Figure 6:**
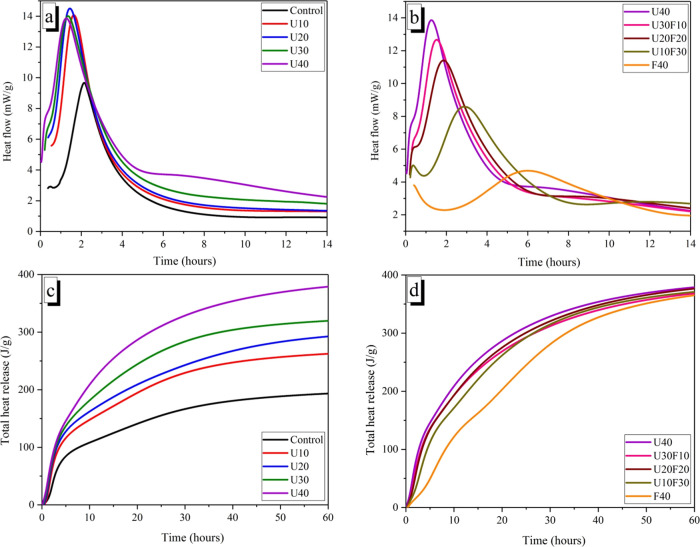
Isothermal calorimetry of the MKPC pastes (normalized to mass of MgO and KH_2_PO_4_) displaying heat flow (a, b) and cumulative heat flow (c, d) for varying UFA additions and varying ratio of UFA:FA fixed at 40 wt %.

Figure 6 shows the hydration heat release curves (normalized to mass of MgO + KH_2_PO_4_) of the MKPC paste with different formulations. With the incorporation of increasing UFA content, the occurrence of a second exothermic hydration peak (associated with crystallization) gradually emerged at an earlier time. For example, the maximum heat flow decreased from 1.62 to 1.27 h when the UFA replacement ratio increased from 10 to 40 wt %, which indicates that the addition of UFA accelerated the hydration reaction of MKPC pastes. However, in the UFA:FA system, the maximum heat flow was delayed from 1.27 to 6.12 h when the FA replacement ratio increased from 0 to 40 wt %, respectively. This corresponds to the setting time observations in Figure 2. This trend is attributed to the smaller UFA particles providing a greater number of nucleation sites to encourage the formation of hydration products, which can shorten and accelerate the heat evolution. Conversely, the incorporation of FA particles reduces the hydration exotherm of MKPC pastes due to the dilution effect of larger FA particles.^35^

In addition, the total exothermic curves of MKPC (normalized to mass of MgO + KH_2_PO_4_) are plotted in Figure 6. The exotherm per gram of MgO + KH_2_PO_4_ increases significantly with increasing UFA incorporation (Figure 6c), indicating the occurrence of potentially different reactivities or secondary reactions associated with the UFA addition. Based on previous studies,^9^ it is postulated that the aluminosilicate glass present in UFA reacts chemically with phosphoric acid, which suggests that an aluminum (or aluminosilicate) phosphate phase is likely to be formed in the MKPC matrix as a minor secondary phase. Further work would be required to provide supporting evidence for the secondary phase(s) formed. When the UFA/FA incorporation was fixed at 40 wt %, the total heat release per gram of MgO + KH_2_PO_4_ was slightly higher for the U40 sample compared to the F40 sample, indicating that the reactivity of UFA was higher than that of FA.

### Phase Assemblage

**Figure 7:**
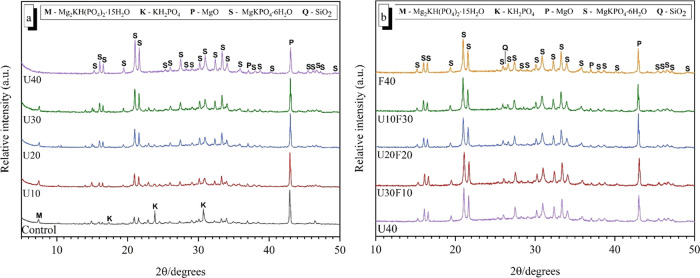
Powder diffraction patterns for MKPC pastes at 7 days of curing: (a) increasing UFA additions and (b) varying ratio of UFA:FA at 40 wt % addition.

**Figure 8:**
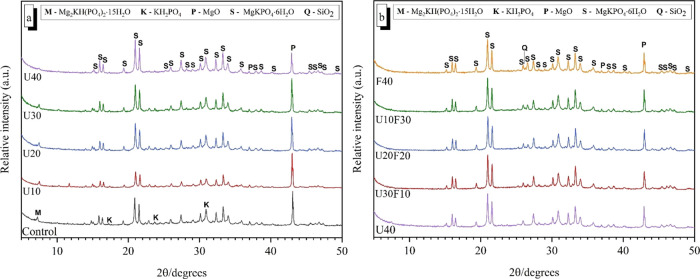
Powder diffraction patterns for MKPC pastes at 28 days of curing: (a) increasing UFA additions and (b) varying ratio of UFA:FA at 40 wt % addition.

XRD patterns of the hardened MKPC binders cured for various ages (7 and 28 days) are shown in Figures 7 and 8. MgKPO_4_·6H_2_O (PDF #00-020-0685) was the main crystalline product identified within all blended MKPC binders, in addition to traces of unreacted periclase (MgO, PDF #00-001-1235). After 7 days of curing, when the addition of UFA was below 30 wt % (Figure 7a), an additional reaction product, Mg_2_KH(PO_4_)_2_·15H_2_O (PDF #00-044-0790), was identified in the samples as detailed elsewhere.^43,44^ In the pure MKPC binder (control), in addition to MgKPO_4_·6H_2_O, Mg_2_KH(PO_4_)_2_·15H_2_O, and unreacted MgO, strong reflections were observed for unreacted KH_2_PO_4_ (PDF #01-079-0585) after 7 days of curing indicative of incomplete reactions. However, when the addition of UFA and UFA:FA was above 30 wt % (Figure 7b), no Mg_2_KH(PO_4_)_2_·15H_2_O reflections were observed. In contrast, strong MgKPO_4_·6H_2_O reflections were observed after 7 days of curing.

After 28 days of curing (Figure 8a), the relative intensity of the MgKPO_4_·6H_2_O reflections in the MKPC control all increased in conjunction with an observed decrease in the relative intensity of the MgO reflections, suggesting that the content of MgKPO_4_·6H_2_O phase increased with curing time. However, it is worth noting that the weak Mg_2_KH(PO_4_)_2_·15H_2_O reflections can still be observed.

The relative intensities of the MgKPO_4_·6H_2_O reflections for the UFA:FA 40 wt % blends in Figure 7b were significantly greater than the UFA samples below 30 wt % in Figure 8, which indicates that MgKPO_4_·6H_2_O was present at a higher fraction in the UFA:FA blends. This leads to the development of MgKPO_4_·6H_2_O phase, which was attributed to the observed increased strength gain at 28 days (Figure 3). It is postulated that the low compressive strength of the sample with UFA below 30 wt % was due to the large amount of the poorly crystalline Mg_2_KH(PO_4_)_2_·15H_2_O phase in the MKPC matrix, implied due to the relatively low intensity of the MgKPO_4_·6H_2_O reflections.^43^

### Thermal Analysis

**Figure 9:**
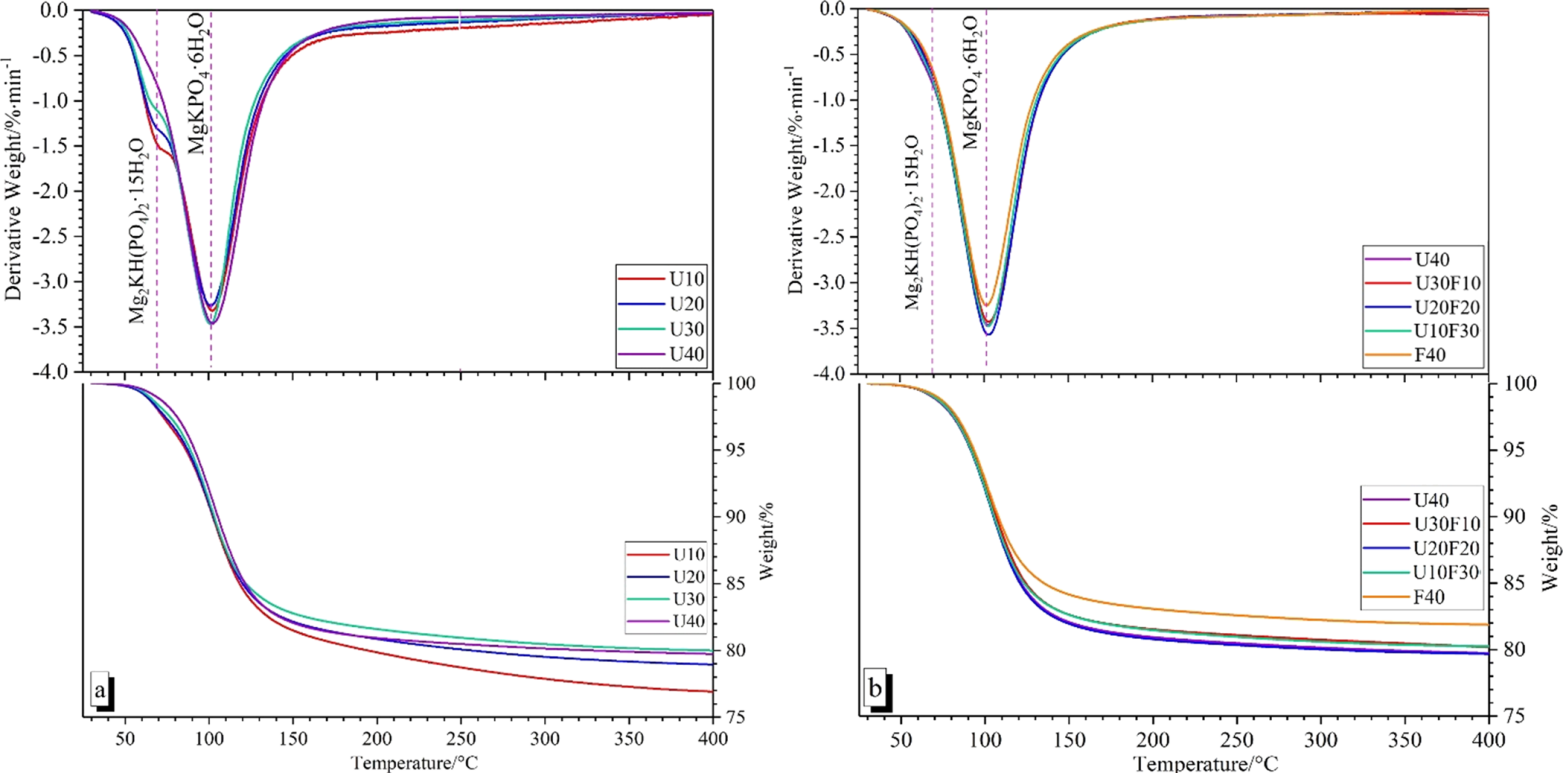
DTG curves for MKPC pastes at 28 days of curing: (a) increasing UFA additions and (b) varying ratio of UFA:FA at 40 wt % addition.

Thermogravimetric analysis (TGA) of the MKPC paste with various formulations is illustrated in Figure 9. The first weight loss event occurs at ∼70 °C (Figure 9), which represents the dehydration of Mg_2_KH(PO_4_)_2_·15H_2_O (eq 3) in agreement with data reported by Xu *et al.*(44) This weight loss only appeared in the MKPC samples of U10, U20, and U30 wt %, concurring with the XRD phase assignments illustrated in Figures 7 and 8. The main weight loss peak was centered at ∼110 °C, which represented the dehydration of MgKPO_4_·6H_2_O that occurs in a single step leading to the formation of MgKPO_4_, according to eq 4.^20^ In addition to the main dehydration of MgKPO_4_·6H_2_O, a broad weight loss peak at ∼250 °C was also observed in the pure MKPC paste (Figure 8a), which was assigned to unreacted KH_2_PO_4._^44^ There were no significant trends observed to differentiate between the inclusion of UFA and UFA:FA within hardened MKPC binders. Therefore, formulation optimization should be based on the physical properties, phase assemblage, and microstructure.
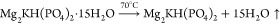
3
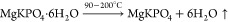
4

### Microstructure

**Figure 10:**
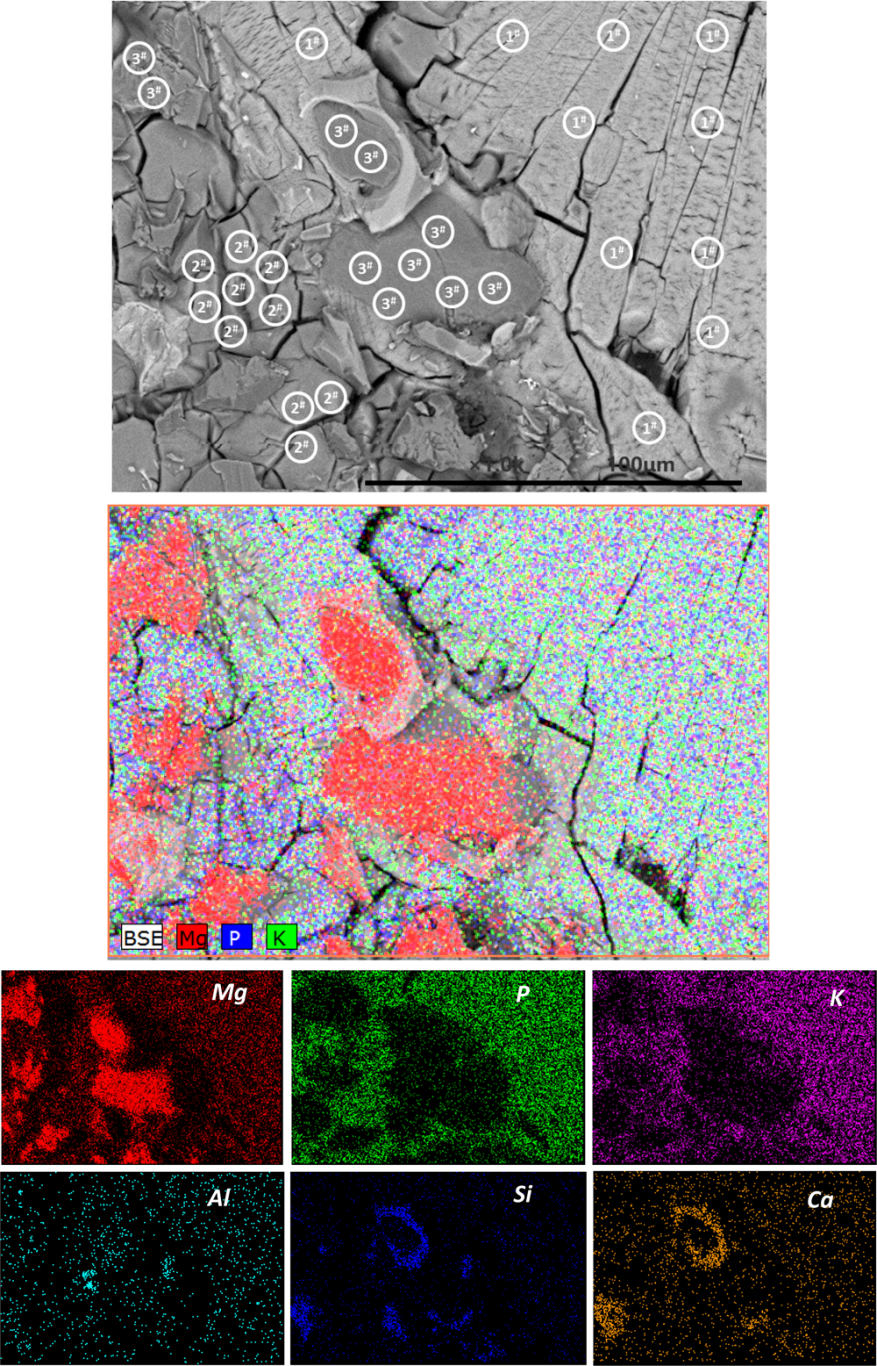
Backscattered electron micrograph and elemental maps of hardened MKPC paste without UFA or FA (control) after 28 days of curing.

**Figure 11:**
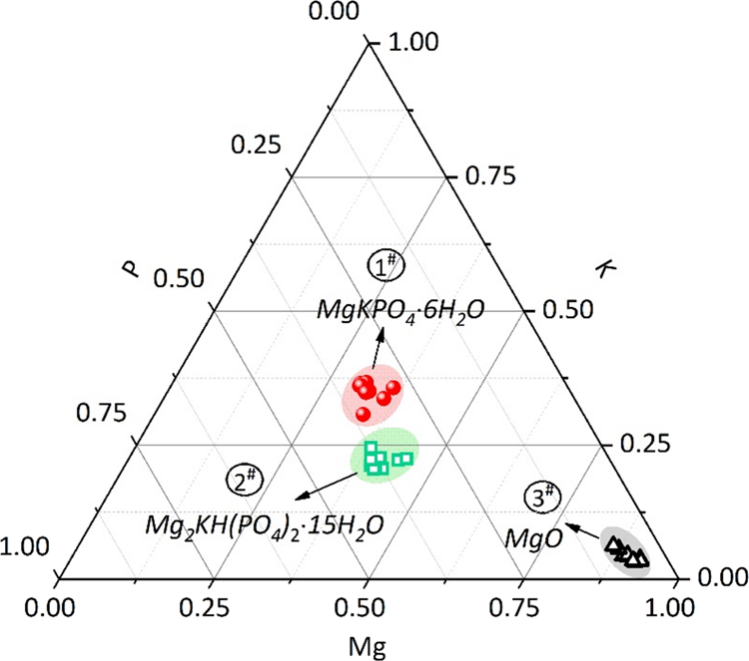
EDX analysis results of hardened MKPC paste without UFA or FA (control) after 28 days of curing, locations of EDS point analysis are marked in Figure 9.

Figure 10 shows the backscattered electron micrograph and elemental maps of hardened MKPC paste without UFA or FA (control) after 28 days of curing. A wide distribution of Mg, P, and K throughout the sample was observed in the EDX maps, consistent with the formation of hydration products. Unreacted MgO particles (dark gray) embedded within the main MgKPO_4_·6H_2_O binder were identified, consistent with XRD data in Figure 7. Traces of impurities (Si and Ca) were also observed around the unreacted MgO (4.3 wt % Si and 2.1 wt % Ca, Table 1). In order to identify the composition of different morphologies in Figure 10, EDX spot analysis was performed in the marked areas 1#, 2#, and 3# using 10 analyses per region. The obtained Mg, P, and K molar ratio is presented as a ternary phase diagram (Figure 11). The results obtained by statistical analysis showed that the elemental ratios of Mg/K and P/K were 0.93 ± 0.09 and 0.94 ± 0.09 for area 1#, 1.86 ± 0.15 and 1.70 ± 0.15 for area 2#, and 21.74 ± 4.84 and 1.39 ± 0.19 for area 3#, respectively. As expected, the EDX points of 1# contained Mg, P, and K with the molar ratios close to the theoretical value of MgKPO_4_·6H_2_O, which has a prism-like crystal morphology with a relatively large length–diameter ratio. This typical morphology was in agreement with previous studies.^10,23^ All the investigated points in area 2# contained Mg, P, and K with the molar ratios close to the theoretical value of Mg_2_KH(PO_4_)_2_·15H_2_O. It was observed that Mg_2_KH(PO_4_)_2_·15H_2_O exists as a short plate-like crystal with an irregular shape, in agreement with previously observations *via* XRD and SEM.^45^ The investigated points in area 3# were assigned to unreacted MgO, which was evident in the EDX maps (Figure 10, Mg).

**Figure 12:**
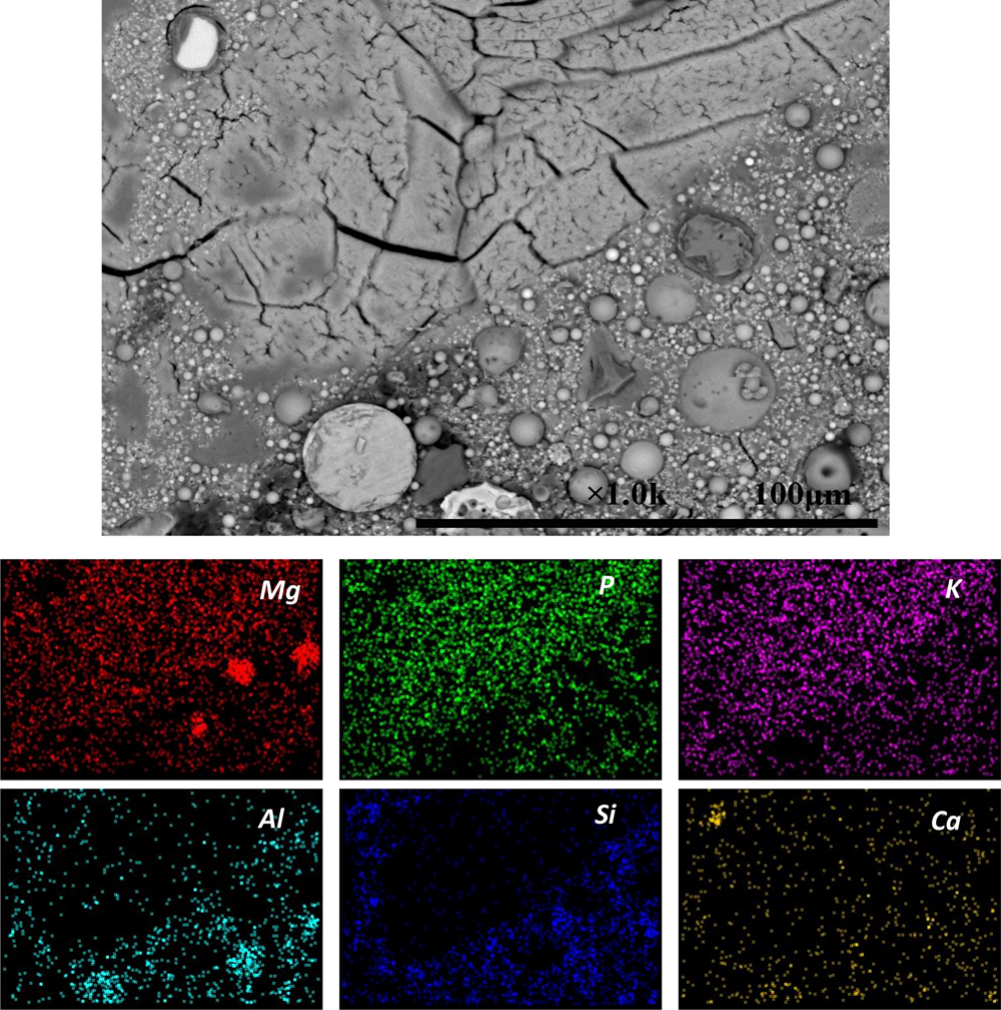
Backscattered electron image and elemental maps of hardened MKPC paste with U20F20 at 40 wt % addition after 28 days of curing.

The SEM/EDX of hardened MKPC paste containing 20 wt % UFA and 20 wt % FA (U20F20) after 28 days of curing (Figure 12) revealed a large number of spherical particles embedded around the prism-like crystal morphology assigned to MgKPO_4_·6H_2_O.^10^ The size of these spherical particles differed greatly, in agreement with the particle size distribution shown in Figure 1. The larger particles are associated with FA while the majority of the smaller spherical particles are associated with UFA. In addition to the large crystallites, MgKPO_4_·6H_2_O also exists within the bulk matrix as a finer structure in which UFA and FA particles are embedded. This variety of particle sizes available allow for the infilling of fine porosity resulting in a dense microstructure.

**Figure 13:**
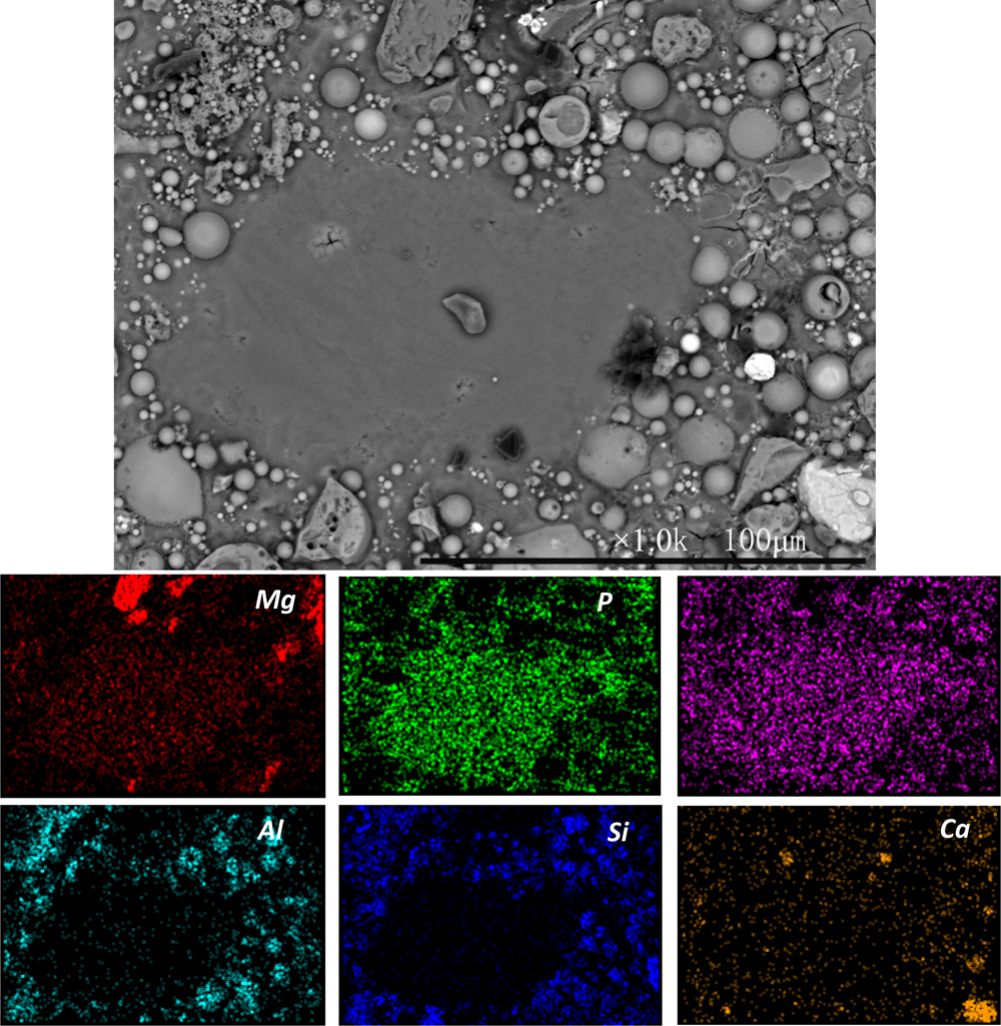
Backscattered electron micrograph and elemental maps of hardened MKPC paste with 40 wt % FA (F40) after 28 days of curing.

Figure 13 shows the SEM/EDX of hardened MKPC paste with 40 wt % FA (F40) after 28 days of curing. Typical spherical morphology of FA and unreacted MgO grains were identified embedded in the MgKPO_4_·6H_2_O matrix. Compared to the U40, the particle size of FA is greater in UFA, consistent with the PSD results shown in Table 1. FA in this study mainly performs a physical filling role in the MKPC matrix, improving the microstructure of MKPC, thereby significantly enhancing the mechanical properties of MKPC (Figure 3).^46^

### MIP

**Figure 14:**
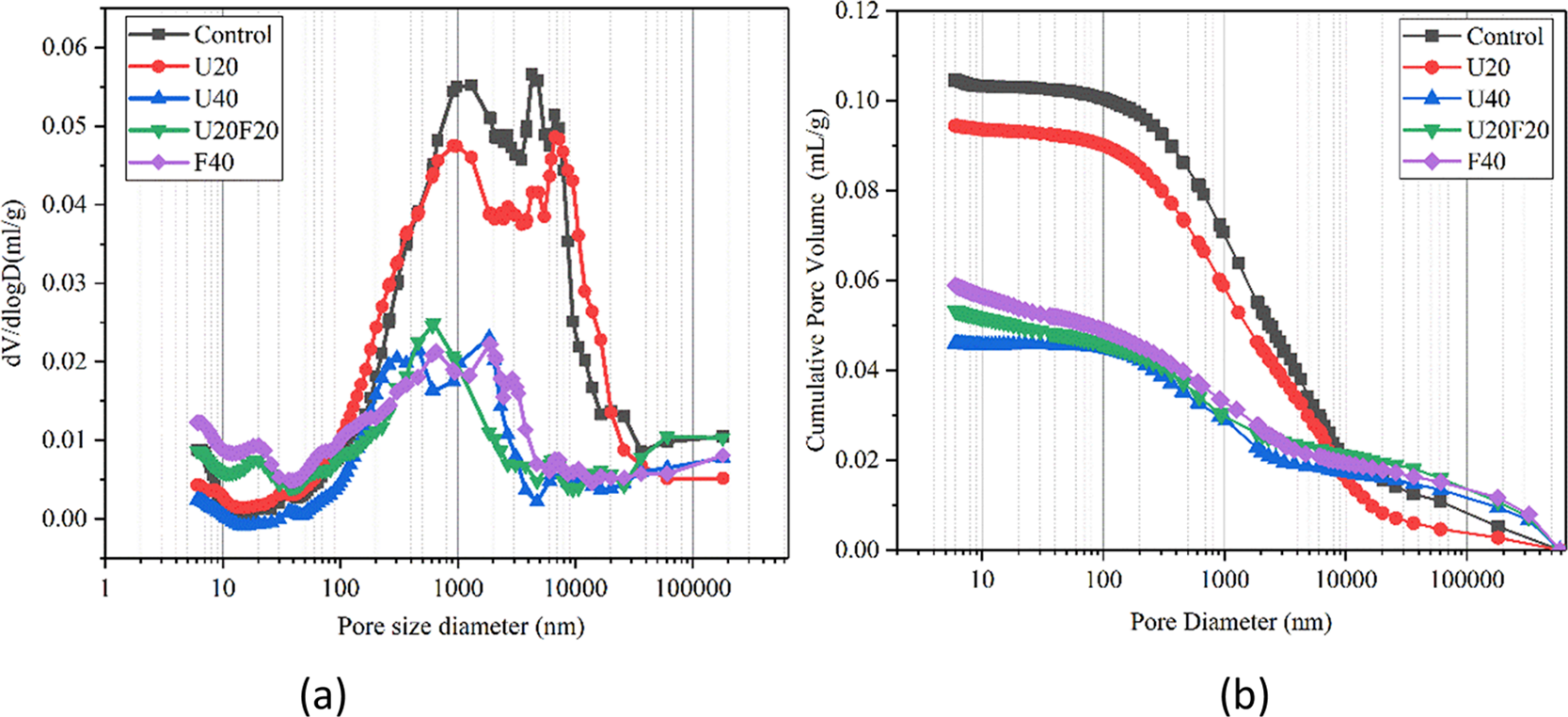
Porosity characterization from MIP: (a) logarithmic differential pore volume distribution; (b) cumulative pore volume.

Figure 14 demonstrates the effect of UFA and UFA:FA composite additions on the pore structure of hardened MKPC samples cured for 28 days, and a summary of MIP porosity measurements are reported in Table 4. The total porosity of MKPC decreased from 21.4% to 8.99% when the UFA replacement addition increased from 0 to 40 wt %. This means that the addition of UFA can improve the pore structure of MKPC significantly,^39^ which was in good agreement with the SEM results and expected filler effect. Compared to UFA, the blended addition of UFA:FA also reduced the total porosity of MKPC. For the U20F20 sample, the total porosity of U20F20 was 9.02%, which is much lower than the MKPC control sample (21.4%) and slightly lower than the UFA/MKPC sample at 40 wt % (10.88%). One explanation for the improved performance of the U20F20 sample could be associated with achieving the highest compressive strength after 7 days of curing. Although not measured, it is expected that the U10F30 sample, which had the highest compressive strength after 28 days of curing, will also have a comparable porosity to the U20F20 MKPC binder.

**Table 4:** Summary of MIP Porosity Measurements

sample	porosity (%)	total mercury intrusion volume (mL/g)
control	21.40	0.1046
U20	18.65	0.0944
U40	8.99	0.0460
U20F20	9.02	0.0533
F40	10.88	0.0589

### NMR

**Figure 15:**
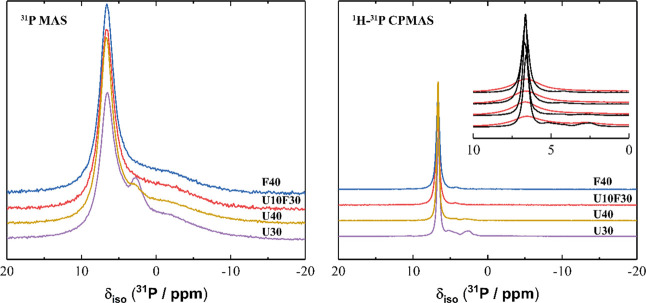
(a) ^31^P MAS (B_0_ = 11.7 T, ν_R_ = 12.5 kHz) NMR and (b) ^1^H-^31^P CPMAS (B_0_ = 11.7 T, ν_R_ = 12.5 kHz, Hartmann–Hahn contact period *t* = 2.0 ms) NMR spectra for selected MKPC pastes at 7 days of curing as marked. For clarity, the vertical axis is scaled by a factor of 10 when comparing panels (a) and (b). The inset in panel (b) shows a direct comparison of the ^31^P MAS (red) and ^1^H-^31^P CPMAS (black) NMR data.

**Figure 16:**
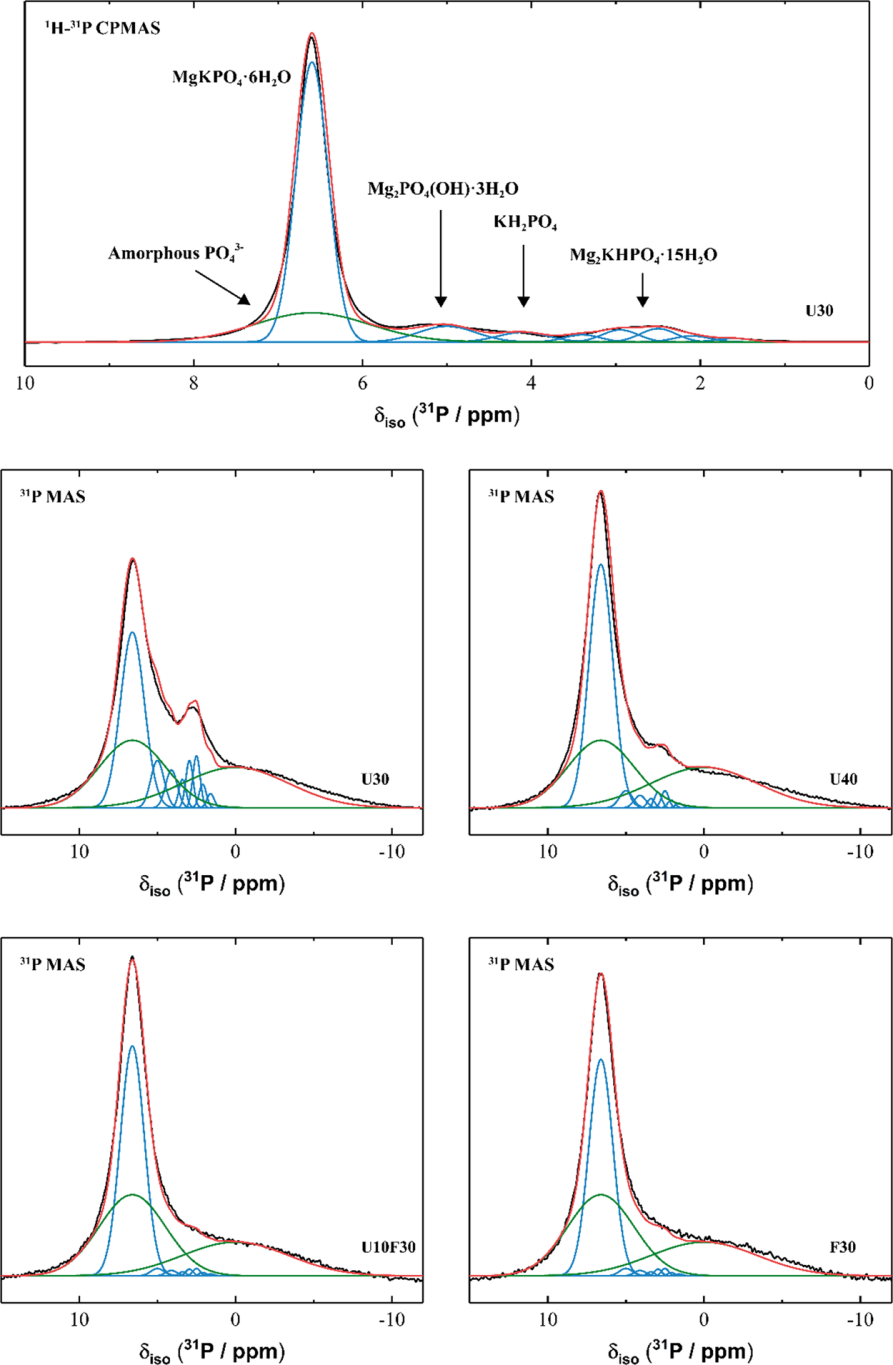
^31^P MAS (B_0_ = 11.7 T, ν_R_ = 12.5 kHz) NMR and ^1^H-^31^P CPMAS (B_0_ = 11.7 T, ν_R_ = 12.5 kHz, Hartmann–Hahn contact period *t* = 2.0 ms) NMR data and associated spectral deconvolutions for selected MKPC pastes at 7 days of curing as marked. In each case, the fit (red line) is the sum of the deconvoluted peaks (blue lines indicate resonances from crystalline phases, green lines indicate resonances from amorphous phases). The data are shown in black.

The ^31^P MAS NMR and ^1^H-^31^P CPMAS NMR spectra for all samples (Figure 15) exhibit a high intensity resonance at δ_iso_ = 6.5 ppm, indicating that all samples comprised primarily MgKPO_4_·6H_2_O,^47^ and a very low intensity resonance at δ_iso_ = 3.8 ppm, indicating the presence of trace amounts of remnant unreacted KH_2_PO_4_.^47^ Samples containing UFA also exhibited a small resonance at δ_iso_ = 3.0 ppm (which can be deconvoluted into four components) assigned to the presence of a small amount of Mg_2_KH(PO_4_)_2_·15H_2_O^47^ as well as a low intensity resonance at δ_iso_ = 5.2 ppm, suggesting the presence of Kovdorskite (Mg_2_PO_4_(OH)·3H_2_O), which has previously been observed in small amounts in magnesium phosphate cements containing struvite, MgNH_4_PO_4_·6H_2_O.^47,48^ This agrees well with the XRD data discussed above. These assignments are consistent with the variation in intensities of each resonance observed in the ^31^P MAS NMR and ^1^H-^31^P CPMAS NMR data, with resonances due to P in phases with greater hydration exhibiting a greater increase in relative intensity in the ^1^H-^31^P CPMAS NMR data. Spectral deconvolution (Figure 16) identifies the presence of a broad resonance exhibiting a distribution of δ_iso_, overlapping the main resonance at δ_iso_ = 6.5 ppm in the ^31^P MAS NMR data for all samples, suggesting the presence of P within an amorphous phase comprising orthophosphate environments,^49−51^ similar to that previously observed in magnesium phosphate cements.^47^ Another broad resonance exhibiting a distribution of δ_iso_ is observed at δ_iso_ = 1.5 ppm in the deconvoluted ^31^P MAS NMR data for all samples (Figure 16), suggesting the presence of P within an amorphous phase comprising pyrophosphate environments.^52^ The significant reduction in intensity of the resonances due to these amorphous phases in the ^1^H-^31^P CPMAS NMR spectra compared with the ^31^P MAS NMR suggests that they each exhibit a low degree of hydration, consistent with their amorphous nature.

**Figure 17:**
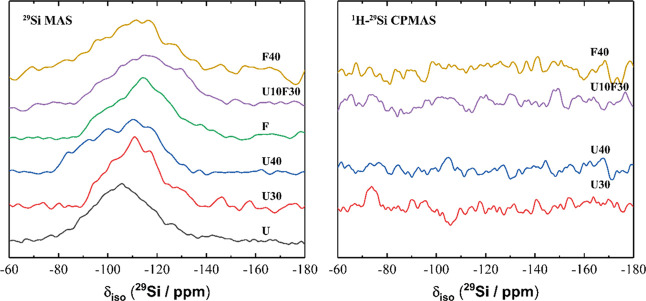
(a) ^29^Si MAS (B_0_ = 11.7 T, ν_R_ = 12.5 kHz) NMR and (b) ^1^H-^29^Si CPMAS (B_0_ = 11.7 T, ν_R_ = 12.5 kHz, Hartmann–Hahn contact period *t* = 1.7 ms) NMR spectra for selected MKPC pastes at 7 days of curing as marked. (Note: U is ultrafine fly ash, F is fly ash.)

^29^Si MAS NMR spectra for each sample (Figure 17) exhibited a broad resonance at δ_iso_ = −110 ppm and −115 ppm for samples containing UFA and FA, respectively. The line shape and intensity of these resonances were largely consistent with those due to the UFA and FA precursors, comprising primarily Q^4^(0Al) Si sites.^52^ The ^1^H-^29^Si CPMAS NMR data do not show any signal above the noise, indicating an absence of hydrated Si sites present in the magnesium phosphate cement samples. The resonances in the ^29^Si MAS NMR can therefore be attributed solely to anhydrous Si sites in unreacted FA and UFA precursor particles. Slight differences in the line shape and position of the resonances in ^29^Si MAS NMR MAS spectra for the UFA and FA precursors and the magnesium phosphate cement samples are likely due to the line broadening and reduced signal/noise resulting from the significant quantity of Fe_2_O_3_ in the samples rather than dissolution and consumption of any UFA and FA Si sites during reaction. This suggests that Si in FA and UFA does not participate in a reaction during the formation of the magnesium phosphate cements.

**Figure 18:**
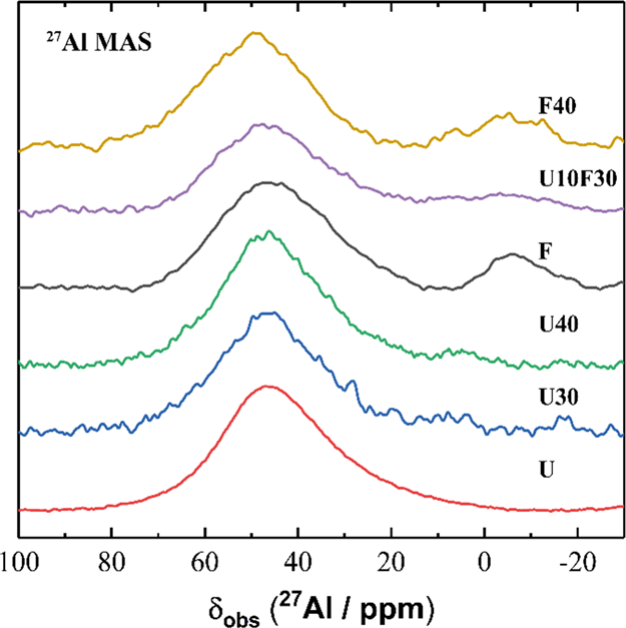
^27^Al MAS (B_0_ = 11.7 T, ν_R_ = 12.5 kHz) NMR spectra for selected MKPC pastes at 7 days of curing as marked. (Note: U is ultrafine fly ash, F is fly ash.)

^27^Al MAS NMR spectra for all magnesium phosphate cement samples and FA and UFA precursors (Figure 18) exhibit a broad tetrahedral Al resonance with maximum intensity at δ_obs_ = 47 ppm. A broad octahedral Al resonance is observed at δ_obs_ = −5 ppm in the ^27^Al MAS NMR spectra for the FA precursor and the MKPC samples containing FA. These resonances were assigned to a single disordered tetrahedral AlO_4_ site and a single disordered octahedral AlO_6_ site, consistent with previous observations for fly ashes.^52^ There was no change in Al speciation upon the acid–base reaction of MKPCs, indicating that Al contained within FA and UFA did not participate in the reaction, consistent with ref (23). Together with the ^29^Si MAS and ^1^H-^29^Si CP MAS NMR data, this clearly demonstrated that in this study, FA and UFA acted primarily as a diluent, leading to rheological and mechanical improvements, with no evidence of secondary reaction between MKPC and FA/UFA.^53^ However, the effect of UFA/FA additions was found to lengthen the total reaction time of MKPC blends, suggesting that secondary reactions may be occurring or altering the curing process of MKPC.

Previous work undertaken by some of the authors discovered the formation of a potassium aluminosilicate secondary phase *via*^27^Al, ^29^Si, and ^39^K MAS NMR in MKPC binders that were blended with FA or slag,^9,54^ which was not replicated in this study. This suggests that if secondary reactions have occurred, they are minor and may more likely represent a modification to the extended curing (i.e., development of the interconnectivity of MKPC). In particular, the authors found that the ^27^Al MAS NMR revealed that the Al^IV^ band was slightly deshielded compared to unreacted FA (among other changes). This finding is not widely observed by other MKPCs researchers, which is attributed to the different formulation design, such as various M/P molar ratio, water/binder ratio, FA addition, etc., which could lead to different hydration products formed in the FA/MKPC system.^40^ Another postulation for the difference is the extended curing time. A recent study revealed that the FA/MKPC binder in question continues to react for up to ∼90–100 h^55^ (compared to ∼60 h in the present study; Figure 5). This extended reaction period allows more time and opportunity for the MKPC precursors and FA to react, however; it should be emphasized that refs (9, 54) do not state that this reaction is widespread but rather a minor secondary phase present within formulations designed specifically for the immobilization of reactive metals present in UK nuclear wastes. It is for these reasons that we believe that there is a difference in the NMR data and present study overall.

## Conclusions

In this study, the effects of UFA and UFA/FA additions on the properties, phase evolution, and microstructure of MKPC were investigated. Based on the presented experimental findings, the following conclusions can be drawn:

(1) Both addition of UFA and FA can prolong the setting time of MKPC paste. Compared with UFA, the addition of FA can increase the setting time of MKPC paste significantly.
(2) Compared with the single addition of either UFA or FA, a composite blend of UFA:FA was found to improve the compressive strength development of MKPC. From SEM/EDX and MIP results, UFA and FA mainly behaved as a filler in the blended MKPC system, with some indications of altering the curing behavior or minor secondary reactions. In the MKPC hardened matrix with UFA/FA, a relatively dense microstructure was observed.
(3) When the replacement of UFA was below 30 wt %, an additional hydrated magnesium phosphate compound, Mg_2_KH(PO_4_)_2_·15H_2_O, was observed by XRD, SEM, TGA, ^31^P MAS NMR, and ^1^H-^31^P CP-MAS NMR. However, no Mg_2_KH(PO_4_)_2_·15H_2_O was detected when the replacement of UFA or UFA:FA was higher than 40 wt %.
(4) Overall, the successful addition of UFA was achieved into blended MKPC binders, and based on the properties investigated, it is possible to state that the optimized formulation was the U10FA30 addition. This resulted in the highest compressive strength, highest workable paste with a dense microstructure. The advantages of including UFA could be high specific surface area and better filling effect.
